# Metamorphism in a wet Martian middle crust

**DOI:** 10.1073/pnas.2416680121

**Published:** 2024-11-22

**Authors:** Richard Palin, Jon Wade, Brendan Dyck

**Affiliations:** ^a^Department of Earth Sciences, University of Oxford, Oxford OX1 3AN, United Kingdom; ^b^Department of Earth, Environmental and Geographical Sciences, University of British Columbia, Vancouver, BC V1V 1V7, Canada

The article by Wright et al. (1) is the latest in a series of papers suggesting that Mars’ inventory of liquid water was sequestered into the crust, rather than entirely lost to space (2, 3). The authors constrain the presence of water in the mid crust (~11.5 to 20 km depth) by comparing bulk-rock geophysical data obtained from the InSight lander with a petrophysical model using a linear combination of putative igneous compositions (mafic basalt to felsic anorthosite). Using these parameters, the “best fit” to the InSight data is a water saturated (*γ_w_* = 100%) mid-crust. Although performing an inversion to determine physical properties from seismic data is an appropriate approach, the geological assumptions upon which it is based are less sound.

Martian areotherms indicate a minimum increase in temperature with depth of 12 °C/km, but potentially as high as 20 °C/km (4). A median value of 16 °C/km equates to mid-crustal temperatures of ~180 to 320 °C, placing the equilibrated conditions firmly within the metamorphic domain (Fig. 1). Terrestrial field studies (5), laboratory analyses (6), and petrological modeling (2, 4) show that hydrated igneous rocks at such conditions transform to minerals characteristic of the prehnite-pumpellyite (PP) or pumpellyite-actinolite (PA) facies (Fig. 1). When hydrated and metamorphosed to PP or PA conditions, mafic rocks (e.g., the basalt endmember) transform to assemblages of chlorite + actinolite + albite + silica, with or without laumontite, pumpellyite, prehnite, or serpentine/talc. By contrast, felsic rocks, such as the anorthosite endmember, transform to quartz + albite + prehnite + chlorite or quartz + albite + pumpellyite + chlorite, with or without zeolite or clay minerals (7). Given the ambient conditions of the Martian crust, igneous rocks in long-term contact with water will undergo low-grade metamorphism and hydration, and develop notably different mineral assemblages to those considered by Wright et al. for their inversion. Here, we used the Theriak-Domino petrological modeling program (8) to calculate bulk-rock densities for a hydrous Martian basalt [Fastball (9)] at depth within the Martian middle crust. At 250 °C and 1.5 kbar, the equilibrium assemblage phlogopite, chlorite, actinolite, wollastonite, microcline, and analcime has a bulk density of 2,613 kg/m^3^, matching the target value used by Wright et al. of 2,589 ± 157 kg/m^3^.

**Fig. 1.**
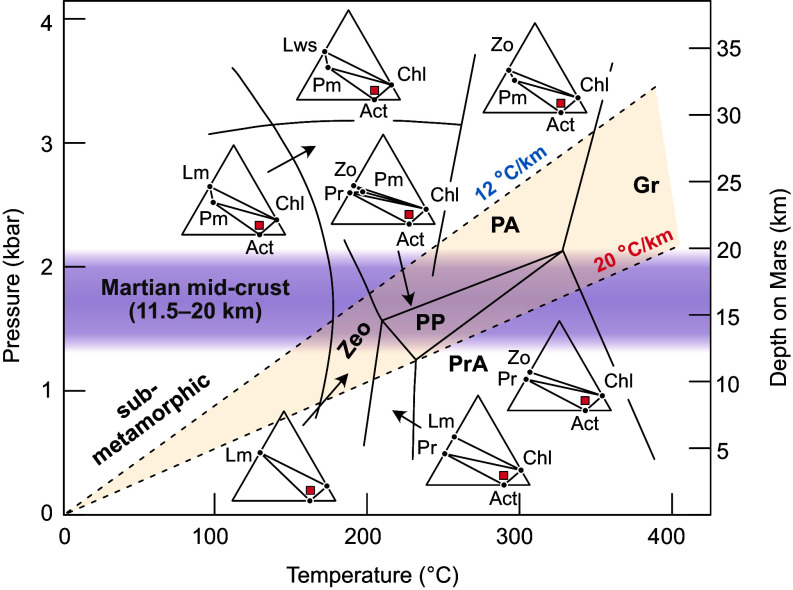
Petrogenetic grid showing metamorphic mineral assemblages stable across Martian pressure–temperature–depth ranges relevant to the work of Wright et al. [modified after (4)]. Zeo—zeolite facies; PrA—prehnite actinolite facies; PP—prehnite pumpellyite facies; PA—pumpellyite actinolite facies; GR—greenschist facies. Mineral assemblages are indicated by ACF diagrams and abbreviations are actinolite (Act), chlorite (Chl), laumontite (Lm), lawsonite (Lws), pumpellyite (Pm), prehnite (Pr), and zoisite (Zo). The red square represents the chemical composition of Martian basalt (9).

Field, experimental, and laboratory-based observations (5–7) show that pristine igneous rocks cannot coexist with hot, reactive aqueous fluids for extended periods of time without undergoing mineralogical change. As Mars’s surface water must have been sequestered underground by the Late Hesperian–Early Amazonian (10), the upper and middle crust has likely existed in a hydrated state for at least 3 billion years, which is ample time to drive PP and PA facies mineralogical transformation. As such the results obtained by Wright et al. from their inversions represent that of an unrealistic pristine igneous Martian middle crust and cannot be used to constrain values for *γ_w_* without accounting for metamorphic reactions.

## References

[1] V. Wright, M. Morzfeld, M. Manga, Liquid water in the Martian mid-crust. Proc. Natl. Acad. Sci. U.S.A. **121**, e2409983121 (2024). 39133865 10.1073/pnas.2409983121 PMC11363344

[2] J. Wade, B. Dyck, R. M. Palin, J. D. Moore, A. J. Smye, The divergent fates of primitive hydrospheric water on Earth and Mars. Nature **552**, 391–394 (2017). 29293210 10.1038/nature25031

[3] E. Scheller, B. Ehlmann, R. Hu, D. Adams, Y. Yung, Long-term drying of Mars by sequestration of ocean-scale volumes of water in the crust. Science **372**, 56–62 (2021). 33727251 10.1126/science.abc7717 PMC8370096

[4] H. Y. McSween Jr., T. C. Labotka, C. E. Viviano-Beck, Metamorphism in the Martian crust. Meteorit. Planet. Sci. **50**, 590–603 (2015).

[5] E. A. Zen, Prehnite-and pumpellyite-bearing mineral assemblages, west side of the Appalachian metamorphic belt, Pennsylvania to Newfoundland. J. Petrol. **15**, 197–242 (1974).

[6] W. E. Seyfried Jr., J. L. Bischoff, Low temperature basalt alteration by sea water: An experimental study at 70 °C and 150 °C. Geochim. Cosmochim. Acta **43**, 1937–1947 (1979).

[7] D. S. Coombs, “Prehnite–pumpellyite facies” in Petrology, A. Buettner, Ed. (Encyclopedia of Earth Science, Springer, Boston, 1989).

[8] C. de Capitani, K. Petrakakis, The computation of equilibrium assemblage diagrams with Theriak/Domino software. Am. Mineral. **95**, 1006–1016 (2010).

[9] S. W. Squyres , Pyroclastic activity at Home Plate in Gusev Crater, Mars. Science **316**, 738–742 (2007). 17478719 10.1126/science.1139045

[10] E. K. Leask, B. L. Ehlmann, Evidence for deposition of chloride on Mars from small-volume surface water events into the Late Hesperian-Early Amazonian. AGU Adv. **3**, e2021AV000534 (2022).

